# Pre-post data from a cogenerational health outreach program within federally qualified health centers: experiences in the encore intergenerational vaccine corps and assessments of different generations and public issues

**DOI:** 10.1016/j.dib.2025.111783

**Published:** 2025-06-16

**Authors:** Cal J. Halvorsen, Bruna Lopez, K. Megan Collier, Cecily Medved, James Emerman

**Affiliations:** a Brown School at Washington University in St. Louis, MSC 1196-251-46, One Brookings Drive, St. Louis, MO 63130, USA; b Center on Aging & Work at Boston College, 140 Commonwealth Ave, Chestnut Hill, MA 02134, USA; c Unit of Occupational Medicine, Karolinska Institute, C6 Institutet för Miljömedicin, C6 Arbetsmedicin Falkstedt, 171 77 Stockholm, Sweden; d Boston College School of Social Work, 140 Commonwealth Ave, Chestnut Hill, MA 02134, USA; e CoGenerate, P.O. Box 29542, San Francisco, CA 94129, USA

**Keywords:** National and community service, Age diversity, COVID-19, Allophilia, Vaccines

## Abstract

This dataset includes pre- and post-service responses from 175 participants in an intergenerational health outreach program within Federally Qualified Health Centers (FQHCs) in the San Francisco Bay Area called the Encore Intergenerational Vaccine Corps (“Vaccine Corps”). The primary sponsor of the Vaccine Corps was AmeriCorps Seniors, a U.S. federal agency that promotes national and community service for Americans aged 55 and older. Response dates cover May 2021 to April 2022, roughly the timeframe in which this program was implemented. Respondents were mostly female (72 %) and ranged in age from 18 to 81 years (*M* = 51.5, *SD*=18.9) during the first survey and 20 to 80 years (*M* = 55.5, *SD*=18.6) in the second survey. Volunteers, who worked to increase COVID-19 vaccine awareness and administrations, were evenly split between identifying as having medical skills (50 %; e.g., being a current or retired nurse or doctor) or being lay volunteers without medical skills (50 %). Questions focused on participants’ assessments of individuals from different generations (i.e., respondents aged 50+ were asked about younger people, and vice versa) and public issues (e.g., adequacy of resource allocation to FQHCs), as well as their experiences in the program. All survey responses were collected online, and survey outreach was conducted by CoGenerate (formerly called Encore.org), the lead program operator of the Vaccine Corps. The complete dataset with 145 variables (3 of which are string/text variables from open-ended responses) is available both as a Stata .do file as well as CSV files. A codebook is available. This dataset is a rare publicly available resource that includes questions related to allophilia (liking of people in the outgroup). It asks respondents about people of different ages, as well as thoughts about and experiences within FQHCs. It can be used to assess the potential for intergenerational programs within public health settings and the experiences of participants within an intentionally intergenerational program. The questions from the survey can also be used or adapted when assessing volunteers’ experiences in similar programs and how respondents view people from different generations.

Specifications Table

**Table:** 

Subject	Social Sciences / Sociology
Specific subject area	Intergenerational service (similar terms: cogenerational and multigenerational service) and civic engagement (similar terms: national and community service, volunteering).
Type of data	.csv file (dataset with numbers) .csv file (dataset with labels) .dta file (Stata dataset) .pdf file (codebook)
Data collection	The pre- and post-surveys were fielded between May 2021 and April 2022 via an online questionnaire. Respondents were volunteers for a San Francisco Bay Area intergenerational volunteer program focused on COVID-19 vaccine awareness and administration. Questions included an adapted version of the Allophilia Scale [1] to assess younger people’s views on older people, and vice versa, as well as newly developed questions related to views on public policy, public health, and social issues. Respondents, who volunteered for this program through a nonprofit organization’s website and provided their contact information in the process, were contacted via email with a request to take these surveys.
Data source location	San Francisco Bay Area
Data accessibility	Repository name: Harvard Dataverse All data can be accessed at the following link: https://doi.org/10.7910/DVN/MKVYLU This archive is hosted by the Harvard Dataverse Network.
Related research article	None

## 1 Value of the Data

• These data, which include a diverse group of volunteers by age, gender, race, language, and medical backgrounds, are valuable for researchers interested in accessing novel measures about the attitudes and perspectives that older people have of younger people, and vice versa, using an adapted version of the Allophilia Scale [1].
• These data are also useful for researchers, social and public sector leaders, policymakers, and program managers due to the inclusion of respondents’ opinions on public policy, public health, and social issues among volunteers in Federally Qualified Health Centers.
• These data cover two points in time between May 2021 and April 2022, earlier and later in volunteers’ time in the intergenerational volunteer program. As such, researchers can use these data to assess change over time.
• These data reflect the views of volunteers who chose to join an intergenerational volunteer program, called the Encore Intergenerational Vaccine Corps, in the San Francisco Bay Area early in the COVID-19 pandemic. As such, these data will be useful to public health researchers and, eventually, historians focused on the views and experiences of volunteers who joined a program to conduct vaccine outreach and administration.
• Researchers are welcome to use the measures in this dataset for their own studies; measures that may be particularly useful include our adapted Allophilia Scale (i.e., how do younger people view older people and vice versa); questions related to views on public policy, public health, and social issues; and questions focused on respondents’ experiences in their volunteer program.

## 2 Background

Federally Qualified Health Centers (FQHCs) provide medical care and services to underserved populations. Due to capacity constraints at FQHCs during the COVID-19 pandemic, including staffing shortages, [2] the nonprofit organization, Encore.org, and the state agency, California Volunteers, partnered with the federal AmeriCorps Seniors agency to develop the Encore Intergenerational Vaccine Corps (“Vaccine Corps”) [3]. The Vaccine Corps, in addition to recruiting volunteers with a mix of medical (e.g., retired nurses and doctors) and non-medical backgrounds, also had a goal to recruit multigenerational, multiracial, and multilingual volunteers who reflected the communities they served. This dataset is the result of a rapid data tracking plan developed in collaboration between a university-based researcher and program developers. This dataset was designed to track the experiences and perceptions of the Vaccine Corps volunteers, both early and later in their volunteer roles, from May 2021 to April 2022. Inspired by the contact hypothesis, [4] the primary goal of this dataset was to track changes in volunteers’ perceptions of members of different generations during an intentionally intergenerational program, measured by an adapted Allophilia Scale [1]. The secondary goal of this dataset was to track changes in perceptions of public policy, public health, and social issues, and the third goal was to assess volunteers’ experiences in the Vaccine Corps.

## 3 Data Description

This dataset contains 145 variables among 175 volunteers in the Vaccine Corps, distributed across two time points. Several sets of identical questions were asked in both time periods. The codebook and data files are available through the Harvard Dataverse [5].

The data are included in three forms, which include a Stata data file with custom variable labels, a CSV file with numbers (e.g., 1 for female, 0 for male), and a CSV file with labels (e.g., female, male). Further, a codebook in PDF format is provided.

Variables include:

• Respondents’ thoughts about younger or older people. Specifically, for volunteers aged 55 years and older, questions asked respondents to what extent they agreed with 19 statements about younger people on a 7-point Likert-type scale from 1 = Strongly disagree to 7 = Strongly agree. Conversely, for volunteers aged 18 to 54 years, questions asked respondents to what extent they agreed with 19 statements about older people on the same scale. Example items include, “In general, I have positive attitudes about younger/older people,” “I feel a sense of belonging with younger/older people,” and “I feel like there is much to learn from younger/older people.”
• Respondents’ agreement with a set of five statements related to COVID-19 vaccinations as well as the adequacy of resources given to FQHCs, programs that support older and younger people, and other topics on a 7-point Likert-type scale from 1 = Strongly disagree to 7 = Strongly agree.
• Respondents’ self-rated likelihood of engaging in paid or volunteer work, advocacy, or other activities intended to benefit people with very low incomes, immigrants, racial and ethnic minorities, older people, and younger people on a 7-point Likert-type scale from 1 = Not at all likely to 7 = Very likely.
• Respondents’ interactions with people outside of their families who are much older and younger than them where they work, volunteer, and socialize, on a 5-point Likert-type scale from 1 = Not at all to 5 = All the time.

Several additional variables include data from the second survey only. These include questions about the service duration, intensity, and schedule; and whether the volunteers helped to administer vaccines during the program and, if yes, their estimates of the total number of vaccines they administered. Volunteers were also asked, given their experiences in the Vaccine Corps, if they would be interested in working with someone of another generation in the future. Further, on a 5-point Likert-type scale from 1 = Not at all to 5 = To a great extent, volunteers were asked to what extent they interacted with volunteers, staff, and community members of different generations; helped in efforts to prevent the spread of COVID-19; and learned more about the work of FQHCs. Finally, on the same 5-point scale, volunteers were asked about their overall satisfaction with components of the Vaccine Corps program itself, including the online application experience, waiting period between the application and assignment to a site, training experience, and volunteer experience.

To aid researchers in their analysis, a set of demographic variables are also provided. These include age (continuous) and age group, gender, race/ethnicity, and languages spoken. A variable that indicates whether the respondent answered questions from the first survey only, the second survey only, or both surveys was also created for this dataset.

In Stata, the variables contain labels that indicate when the question was asked. These are indicated as S0 (when the respondent initially signed up to volunteer for the Vaccine Corps), S1 (the first survey), and S2 (the second survey). The variables have been organized in this order.

Table 1 shows the sample characteristics.

**Table 1:** Sample characteristics (*N* = 175).

	n	%
**Volunteer Type**^a^		
Medical	87	49.7 %
Non-medical	88	50.3 %
**Age**^a^		
18–25	24	13.7 %
26–40	33	18.9 %
41–54	15	8.6 %
55–70	77	44.0 %
71+	26	14.9 %
**Gender**^a^		
Female	120	71.9 %
Male	47	28.1 %
**Race**^a^, ^b^		
White	99	59.6 %
Black	9	5.4 %
Hispanic	12	7.2 %
Asian	54	32.5 %
2+Races	7	4.2 %
**Language abilities**^a^, ^c^		
Spanish	40	23.8 %
Chinese (Mandarin or Cantonese)	13	7.7 %
Another language (not English)	31	18.5 %
At least one language in addition to English	77	45.8 %
**Survey response**		
Survey 1 only	87	49.7 %
Survey 2 only	19	10.9 %
Both surveys	69	39.4 %

Percentages may not add up to 100 % due to rounding.

a Variables measured at time of signing up for the Vaccine Corps (denoted S0 in the labels for these variables in the dataset).

b Respondents could identify more than one race; as such, percentages add up to >100 %.

c The survey was asked only in English; as such, the assumption is that all respondents understood at least basic English. Respondents could identify more than one language.

To protect the anonymity of respondents, we removed a select few variables from this dataset before publishing. These include the volunteers’ names and contact information, as well as if they were veterans or currently in the military (we dropped these last two variables because only four respondents reported being veterans and one reported being in the military). Because several languages were noted by only one or a few respondents (e.g., Tagalog, Korean, Portuguese, Russian), we combined those into one category to increase anonymity; we also combined Mandarin and Cantonese into the larger Chinese category for the same reason.

## 4 Experimental Design, Materials and Methods

This study is an example of an academic-practitioner collaboration that balances the sometimes conflicting needs of both parties [6]. Specifically, this study’s first author worked closely with Vaccine Corps planners to develop a study design that could be trusted (i.e., high academic standards), collected data of interest to program planners and funders (e.g., feelings about people from different age groups and experiences in the program), and did not lead to survey fatigue (i.e., a goal of 7 min or less to respond).

Only people who had signed up to volunteer for the Vaccine Corps from Spring 2021 to Spring 2022 and lived in the San Francisco Bay Area were invited to take the survey. Respondents, who volunteered for this program through the website of the nonprofit organization, CoGenerate (which was then called Encore.org), were contacted via email with a request to take these surveys. The first author of this study served as an advisor to the program planners when drafting the survey instruments. The academic-practitioner team engaged in several conversations and email communications regarding the survey instruments (e.g., what topics to pursue, how to ask quality survey questions, editing of questions, reviewing the online survey forms from computers and smartphones, etc.). The surveys were then emailed to respondents via a Salesforce application: The first survey was sent to volunteers from May to July 2021, with the second survey sent to volunteers from August 2021 to April 2022. Respondents received $25 electronic gift cards for the completion of each survey (i.e., $50 total if both surveys were completed).

The set of 19 questions that asked younger people about older people, and vice versa, were adapted from the Allophilia Scale [1]. The first 17 questions had identical stems to the original Allophilia Scale, which was created to be adapted to whatever the “outgroup” is. That is, the original published article about the Allophilia Scale provided the stem for each prompt followed by an indicator to include the name or description of the group being considered (e.g., “I respect [members of the outgroup]”). As such, for adults between the ages of 18 and 54 years, we added “older people” to each prompt (e.g., “I respect older people”). For adults aged 55 years and older, we added “younger people” to each prompt (e.g., “I respect younger people”). We chose 55 as the age cutoff to correspond with existing national service programs for older adults, such as Senior Companions and Foster Grandparents, which begin at age 55 [7]. In consultation with the Vaccine Corps planners, we also added two more prompts: “I feel like there is much to learn from younger/older people” and “I would like to work with younger/older people.” Using the same answer options as the original Allophilia Scale, respondents could select their level of agreement from 1 = Strongly disagree to 7 = Strongly agree for each prompt. The Cronbach’s alpha values for the adapted Allophilia Scales were high, around 95 % (see Table 2). All other questions were developed specifically for this study and, for pragmatic purposes, were designed solely to have high face validity. All questions are included in the codebook that accompanies this article.

**Table 2:** Cronbach’s alpha values for adapted Allophilia Scales.

Outgroup	Survey	α	n range^a^
Younger people^b^	1	.95	84–89
Younger people^b^	2	.95	55–57
Older people^c^	1	.97	63–67
Older people^c^	2	.96	30–31

a Pairwise computation was used in Stata to determine the alpha level.

b Respondents aged 55 years and older were asked questions about younger people.

c Respondents between the ages of 18 and 54 years were asked questions about older people.

## Limitations

This study is observational in nature; in other words, there is no control group, and only volunteers who chose to respond to our emailed survey requests were included in the dataset.

During data cleaning, we discovered that two people below the age of 55 took the Allophilia Scale questions designed for people aged 55 years and older (one person each in the first and second surveys). These individuals also responded to the Allophilia Scale questions intended for their age group, too. We decided to keep their responses in the dataset, as the values appeared genuine, and this was likely the result of a survey programming glitch.

Perhaps unsurprisingly, we learned through conversations with program implementers that the intergenerational nature of the Vaccine Corps—a key feature supported by the implementing organization, Encore.org—was not the focus for the FQHC staff or volunteers at the time of this study. This is likely due to several factors, including the public health urgency of the COVID-19 pandemic combined with staffing shortages and other issues facing FQHCs at the time of data collection [2].

## Ethics Statement

The study that led to the creation of this dataset was determined to be exempt from review by the Boston College Institutional Review Board. (The study’s first author was based at Boston College during the data collection.) Because the study’s first author was not collecting the data, but rather advising the Vaccine Corps’ planners on study methods and questions, this was determined to be secondary data of minimal risk. However, the Vaccine Corps’ planners regularly communicated with the funder (the federal AmeriCorps agency), who knew about and approved the study. Further, the first author reviewed important human subjects considerations (e.g., keeping data in a secure location and anonymizing data for public reports) with the Vaccine Corps’ planners throughout the study.

## Credit Author Statement

**Cal J. Halvorsen:** Conceptualization, Methodology, Formal analysis, Investigation, Resources, Data curation, Writing – Original draft, Writing – review & editing, Supervision, Project administration. **Bruna Lopez:** Formal analysis, Data curation, Writing – review & editing. **K. Megan Collier:** Formal analysis, Data curation. **Cecily Medved:** Software, Data curation. **Jim Emerman:** Conceptualization, Methodology, Project administration, Funding acquisition.

## Data Availability

DataversePre-post data from the Encore Intergenerational Vaccine Corps: A cogenerational health outreach program within Federally Qualified Health Centers (Original data).
